# Pathophysiological Changes and the Role of Notch-1 Activation After Decompression in a Compressive Spinal Cord Injury Rat Model

**DOI:** 10.3389/fnins.2021.579431

**Published:** 2021-01-28

**Authors:** Xing Cheng, Zhengran Yu, Jinghui Xu, Daping Quan, Houqing Long

**Affiliations:** ^1^Guangdong Provincial Key Laboratory of Orthopaedics and Traumatology, Orthopaedic Research Institute/Department of Spine Surgery, The First Affiliated Hospital, Sun Yat-sen University, Guangzhou, China; ^2^PCFM Lab, GD HPPC Lab, School of Materials Science and Engineering, Sun Yat-sen University, Guangzhou, China

**Keywords:** decompression, CSCI, motor function, pathophysiological changes, Notch-1

## Abstract

Surgical decompression is the primary treatment for cervical spondylotic myelopathy (CSM) patients with compressive spinal cord injury (CSCI). However, the prognosis of patients with CSCI varies, and the pathophysiological changes following decompression remain poor. This study aimed to investigate the pathophysiological changes and the role of Notch-1 activation after decompression in a rat CSCI model. Surgical decompression was conducted at 1 week post-injury (wpi). DAPT was intraperitoneally injected to down-regulate Notch-1 expression. Basso, Beattie, and Bresnahan scores and an inclined plane test were used to evaluate the motor function recovery. Hematoxylin and eosin staining was performed to assess pathophysiological changes, while hypoxia-inducible factor 1 alpha, vascular endothelial growth factor (VEGF), von Willebrand factor (vWF), matrix metalloproteinase (MMP)-9, MMP-2, Notch-1, and Hes-1 expression in the spinal cord were examined by immunohistochemical analysis or quantitative PCR. The results show that early decompression can partially promote motor function recovery. Improvements in structural and cellular damage and hypoxic levels were also observed in the decompressed spinal cord. Moreover, decompression resulted in increased VEGF and vWF expression, but decreased MMP-9 and MMP-2 expression at 3 wpi. Expression levels of Notch-1 and its downstream gene Hes-1 were increased after decompression, and the inhibition of Notch-1 significantly reduced the decompression-induced motor function recovery. This exploratory study revealed preliminary pathophysiological changes in the compressed and decompressed rat spinal cord. Furthermore, we confirmed that early surgical decompression partially promotes motor function recovery may via activation of the Notch-1 signaling pathway after CSCI. These results could provide new insights for the development of drug therapy to enhance recovery following surgery.

## Introduction

Cervical spondylotic myelopathy (CSM) is a degenerative condition of the spine that leads to static and dynamic compression of the spinal cord (Karadimas et al., 2013a). CSM in cases of compressive spinal cord injury (CSCI) results in various pathophysiological alterations, such as neuroinflammation, microvasculature changes, ischemic-hypoxic injury, blood spinal cord barrier (BSCB) destruction, and neuronal cell death (Yu et al., 2011; Hirai et al., 2013; Tetreault et al., 2015; Vidal et al., 2019). Decades ago, it was reported that surgical decompression of the compressed spinal cord can partially recover neurological function in patients and halt CSM progression (Wiberg, 1986; Ishida et al., 1999; Li et al., 2019; Akter et al., 2020). However, our understanding of the physiological and pathological changes following decompression remains poor due to the lack of a reliable animal model of induced CSM. Thus, studies on the pathophysiological changes after decompression may contribute to improved prognoses of patients with CSCI.

In recent years, numerous attempts have been made to establish a CSCI model in different animals, employing methods such as tumor induction (Izumida, 1995), the use of penetrating hydrogels (Yang et al., 2015) or urethane polymers (Kurokawa et al., 2011), the use of spinal hyperostotic mice (twy/twy) (Hirai et al., 2013), and plastic screw implantation (Kanchiku et al., 2001). Among these approaches, hydrogels and polymers are expected be applicable materials that lead to a chronically progressive injury. However, neurological function changes after CSCI have not been reported for the application of polyurethane polymers. Previously, we successfully established a CSCI rat model using a water-absorbable polyurethane polymer sheet (Long et al., 2013), which provided a foundation for exploring pathophysiological changes in the decompressed spinal cord.

Thus far, only four studies have investigated the decompressed state of CSM in animal models (Harkey et al., 1995; Karadimas et al., 2015b; Dhillon et al., 2016; Haddas et al., 2018). Harkey et al. (1995) demonstrated that decompression can improve neurological function and can alter the necrosis and cavitation of spinal cord tissue in a dog model. Karadimas et al. (2015b) reported on an ischemia-reperfusion injury after decompression in a rat model, which could be prevented by treatment with riluzole. Dhillon et al. (2016) suggested that decompression may increase axon growth rates and promote axon sprouting in a rat model. In addition, (Vidal et al., 2017) found that early decompression is important for achieving better neurological outcomes and demonstrated that delayed decompression is associated with increased astrogliosis, microglia activation, and reduced spinal cord blood flow. As few studies have evaluated the decompression state of CSM, further investigations of pathophysiological changes in the decompressed spinal cord are vital to enable the development of comprehensive drug therapy in order to enhance recovery following surgery.

We have found that various changes, including alterations in microvascular density (Long et al., 2014; Cheng et al., 2015), varying degrees of ischemia-hypoxia (Long et al., 2012; Cheng et al., 2019), matrix metalloproteinase (MMP) expression (Cheng et al., 2019), BSCB disruption (Long et al., 2015), and neuron loss (Xu et al., 2017), occured at different time points after chronic CSCI. Structural and cellular changes after decompression, such as the expression of angiogenesis- and BSCB-related factors, have attracted substantial attention in acute and chronic SCI (Ng et al., 2011; Karadimas et al., 2015a; Randi and Laffan, 2017). Nevertheless, the pathophysiological changes occurring in the decompressed spinal cord have not been widely investigated. Increasing evidence suggests that Notch-1 signaling is involved in central nervous system (CNS) diseases and is associated with cell death, angiogenesis, and BSCB function (Fassbender et al., 2011; Cai et al., 2016). By elucidating the physiological and pathological changes and the role of the Notch-1 signaling pathway after decompression in CSCI, new perspectives may be provided for improving the prognosis of CSM patients with decompression. Therefore, based on our previous studies, we further investigated the physiological and pathological changes in the decompressed spinal cord and the effects of Notch-1 pathway inhibition in a rat CSCI model following decompression.

## Materials and Methods

### Animal Studies

The procedures applied in these experiments were approved by the Research Ethics Committee of Sun Yat-sen University and conformed to all relevant regulatory standards (Animal protocol number: 2020458). A total of 73 adult female Sprague–Dawley rats (weighing 250–300 g; aged 28–30 weeks) were included in this study. Ten of these rats were used to evaluate the extent of expansion of the compression material at 1 day post-injury (dpi) (*n* = 3), 1 week post-injury (wpi) (*n* = 3), or 3 wpi (*n* = 4). Thirty-one rats were randomly allocated to the sham (*n* = 10), CSCI (*n* = 10), or CSCI with decompression (CSCI + decompression; *n* = 11) group. Finally, Thirty-two rats were randomly allocated to the CSCI (*n* = 10), CSCI with decompression and control (CSCI + decompression + NC; *n* = 11), or CSCI with decompression and DAPT (CSCI + decompression + DAPT; *n* = 11) group. One rat died in the Sham group and CSCI group respectively, after the initial surgery due to anesthetic accident, One rat in the CSCI group died on the next day after compression surgery. We performed compression surgeries for 76 rats and three of them died. The mortality rate was 3.9%. The rats were housed in groups of 4–5 per cage on a 12/12-h light/dark cycle with free access to food and water.

### Intraperitoneal Injection

DAPT solution (1 μg/μL) was prepared by dissolving DAPT powder (MCE, United States) in 0.01 M phosphate-buffered saline (PBS) containing 5% dimethyl sulfoxide. The DAPT solution (1 mg/kg per rat) and a negative control were intraperitoneally injected using a microinjection syringe (Cheng et al., 2020).

### Compressive Spinal Cord Injury

Rats in the sham and CSCI groups were anesthetized with a cocktail (3 mg/kg) (Guangzhou FISCLAB Environ. Sci-Tech. Co., Ltd., Guangzhou, China) containing ketamine (31.25 mg/kg), xylazine (1.58 mg/kg), and acepromazine (0.31 mg/kg). Following exposure of the C4–C6 spinal process and laminas from the posterior, C5 laminectomy was conducted to access the epidural space. In the CSCI group, a sheet (1 mm × 3 mm × 1 mm) of water-absorbable polyurethane polymer, (1→4)-3,6-anhydro-a-l-galactopyranosyl-(1→3)-β-D-galactopyranan), was implanted into the C6 epidural space in the dorsal region of the spinal cord (Hu et al., 2011). A modified polyurethane membrane (synthesized by isocyanates and polyols) was coated on the surface of the polymer sheet to reduce the expansion speed. Spinal cord compression was gradually achieved through slow expansion of the polymer sheet. In contrast, animals in the sham groups received only the C5 laminectomy, without insertion of the polymer. For surgical decompression, the same anesthesia methods were applied, and all animals were reopened. In this step, the polymer was removed in the decompression group. After surgery, the paravertebral muscle was sutured in layers, and the skin incision was stapled. Animals received a subcutaneous injection of lactated Ringer’s solution (200 μL) immediately after compression or decompression to prevent dehydration. Penicillin G (80 U/g) was intramuscularly injected to prevent infection during surgery, and carprofen (4–5 mg/kg, Rimadyl, Pfizer) was injected subcutaneously 2 days post-surgery for further pain relief as needed. Both compression and decompression surgeries were performed by the same experienced investigator.

### Motor Function Evaluation

To evaluate the motor function recovery after CSCI, the Basso, Beattie, and Bresnahan (BBB) locomotor scale (Basso et al., 1995) and an inclined plane test (IPT) (Rivlin and Tator, 1977) were conducted at 1 dpi and 1, 2, and 3 wpi. BBB scores range between 0 and 21, where a score of 0 reflects complete paralysis and a score of 21 indicates normal locomotion. Lower scores (0–7) denote isolated joint movements with little or no hindlimb movement; intermediate scores (8–13) express intervals of uncoordinated stepping; and higher scores (14–21) express forelimb and hindlimb coordination. For the IPT, rats were placed horizontally on a smooth tilt board. The board was initially placed in a horizontal orientation (0°), and the angle was increased by 5–10° after each attempt. The maximum angle at which the rats remained on the board for 10 s was recorded. Raw BBB score data for the group of CSCI with decompression were used to evaluate the recovery of animals at 3 wpi in comparison with 1 wpi [Recovery BBB score (CSCI): BBB score (3 wpi) – BBB score (1 wpi)]. If the recovery BBB score ≥ standard deviation (S.D.) of the recovery BBB score (CSCI), then the case was determined to have significant recovery. If the recovery BBB score < S.D. of the recovery BBB score (CSCI), then the case was determined to have no recovery. This evaluation was conducted by two investigators blinded to the group assignments.

### Micro-Computed Tomography

To assess the extent of expansion of the compression material after implantation, the cervical spinal cords of rats in different groups were assessed by micro-computed tomography (micro-CT) (ZKKS-MCT-Sharp-II, Guangzhou Zhongkekaisheng Medical Technology Co., Ltd., Guangzhou, China). Animals were anesthetized with an overdose of 80–120 mg/kg intravenous sodium pentobarbital (Guangzhou Fischer Chemical Co., Ltd.) and transcardially perfused with 0.9% saline followed by 4% paraformaldehyde in 0.1 M phosphate buffer. The whole cervical spinal cord was then carefully harvested and fixed overnight with 4% formaldehyde in phosphate-buffered solution. Prior to micro-CT scanning, the tissues surrounding the spine were carefully removed under a microscope. The compression material of each specimen was presented using 3D-Med 4.3 software (Guangzhou ZhongkeKaisheng Medical Technology Co., Ltd., Guangzhou, China).

### Immunohistochemistry

After perfusion, the C5–C7 spinal cord segment was harvested, fixed overnight with 4% formaldehyde in phosphate-buffered solution at 4°C, and then embedded in paraffin. A series of 25-μm-thick spinal cord sections was used for immunohistochemical (IHC) staining. Microwave epitope retrieval was performed before staining. Sections were incubated overnight at 4°C with a mouse monoclonal antibody against hypoxia-inducible factor 1 alpha (HIF-1α) (1:150; ab1, Abcam, Cambridge, United Kingdom), a rabbit polyclonal antibody against Notch-1 (1:200; ab8925, Abcam, Cambridge, United Kingdom), a mouse monoclonal antibody against Hes-1 (1:50; sc-166410, Santa Cruz Biotechnology, Inc., United States), a mouse monoclonal antibody against vascular endothelial growth factor A (VEGFA) (1:200; ab1316, Abcam, Cambridge, United Kingdom), a rabbit polyclonal against von Willebrand factor (vWF) (1:200; ab6994, Abcam, Cambridge, United Kingdom), a rabbit polyclonal against MMP-9 (1:100; ab38898, Abcam, Cambridge, United Kingdom), and a mouse monoclonal antibody against MMP-2 (1:300; ab86607, Abcam, Cambridge, United Kingdom). Subsequently, the sections were sequentially incubated with a ready-to-use DAKO ChemMate EnVision^TM^ kit (K500711; Dako; Agilent Technologies, Inc., Santa Clara, CA, United States) for 30 min at room temperature. Images of each section of the perilesional spinal cord were acquired at 200× or 400× magnification using an XC30 camera mounted on an Olympus microscope (Olympus Corporation, Tokyo, Japan). Three random images at 200× or 400× magnification were also captured for semi-quantitative analysis. The brown area in the sham spinal cord was analyzed, and the background density was excluded to create the standard. Subsequently, the integrated optical density (IOD) was calculated using Image-Pro Plus 6.0 software (Media Cybernetics, Inc., Rockville, MD, United States). Omission of the primary antibody only controls for unspecific staining of the secondary antibodies.

### Hematoxylin and Eosin Staining

Briefly, slides were placed in xylene in a 37°C incubator. The slides were hydrated by passing through the following alcohol concentrations (100, 100, 95, 95, 75, 75%). Hematoxylin staining (Guangzhou Fischer Chemical Co., Ltd.) was applied for 2–5 min. The slides were then washed in ddH_2_O for 5 min or less, until they turned “blue.” The slides were differentiated in 1% acid alcohol (1% HCL in 70% alcohol) for 5 min. After a washing step, the slides were stained in 1% eosin Y (Guangzhou Fischer Chemical Co., Ltd.) for 10 min. Subsequently, the slides were again washed in ddH_2_O for 2–5 min. The samples were dehydrated in alcohol solutions of increasing concentration (75, 75, 95, 95, 100, 100%) and were cleared of xylene. Finally, the slides were mounted with mounting media, and images (20×, 200×, and 400× magnification) were acquired under a light microscope.

### Real-Time Quantitative Polymerase Chain Reaction

Total RNA was extracted from the compressed or decompressed spinal cord. The purity of the RNA was determined, and reverse transcription PCR (PrimeScript^TM^ RT reagent kit) and real-time quantitative PCR (RT-qPCR) (SYBR Premix Ex Taq II) were performed following the manufacturer’s instructions (Takara Biotechnology Co., Ltd., China). The detailed primer sequences used in RT-qPCR are given in Supplementary Table S1. Cycle threshold (CT) values were recorded, and the relative expression of target genes was calculated using the expression 2^–ΔΔCt^ (Hu et al., 2019).

### Statistical Analysis

All data are expressed as the mean with 95% confidence intervals (CI) or Medians with interquartile ranges. Statistical tests were performed using GraphPad Prism 7.0 software (San Diego, CA, United States). The motor function recovery results were analyzed by repeated-measures analysis of variance (ANOVA) to reveal overall group differences and significant changes over time. One-way analysis of variance followed by Tukey’s *post hoc* test was used for comparisons among multiple groups. Statistical significance was set at *p* < 0.05.

## Results

### Expansion of Water-Absorbable Compression Material After Implantation

**FIGURE 1:**
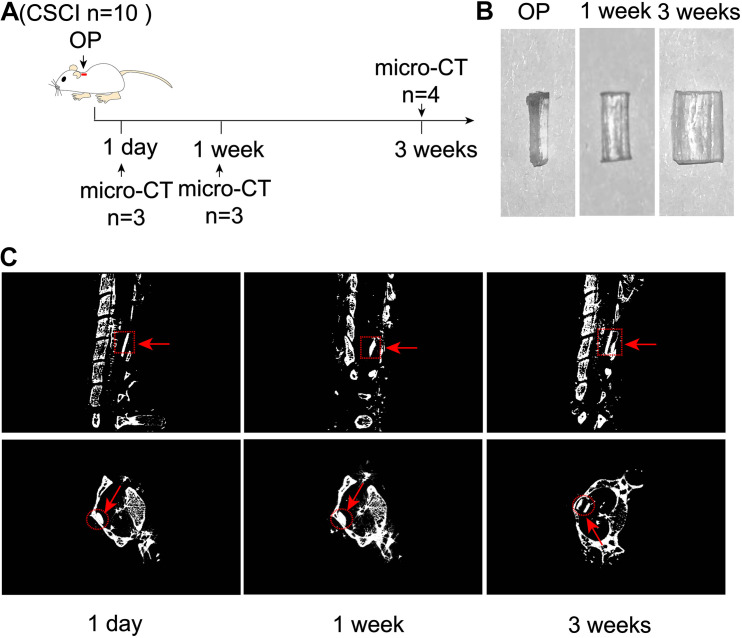
Expansion of compression material after implantation. **(A)** Experimental design. **(B)** Alteration in the water-absorbable polyurethane polymer before and after surgery at 1 and 3 weeks. **(C)** Three-dimensional reconstruction and representative sagittal and transverse micro-CT images of the compressive segment. The red arrow indicates the compression site and material. 1 day post-injury: *n* = 3; 1 week post-injury: *n* = 3; 3 weeks post-injury: *n* = 4.

These experiments were designed to evaluate the extent of expansion of polyurethane polymer sheets under the study conditions (Figure 1A). As shown in Figure 1B, this modified water-absorbable material gradually swelled after implantation and reached a maximal size at 3 wpi. Micro-CT scanning also clearly revealed the extent of expansion at 1 dpi, 1, and 3 wpi (Figure 1C).

### Early Decompression Partially Promoted Motor Function Recovery After CSCI

**FIGURE 2:**
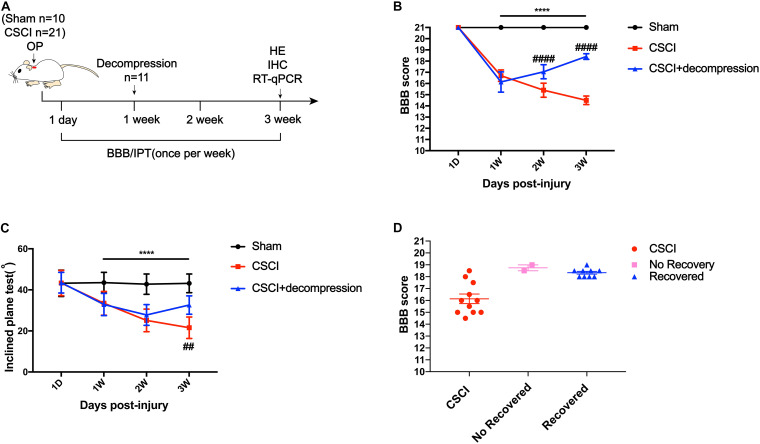
Early decompression promoted motor function recovery after CSCI. **(A)** Experimental design. **(B)** Animals were tested weekly starting from 1 dpi in an open field, and motor function recovery was evaluated according to the BBB score [Two-way ANOVA, *F*(1,8) = 66.13, *p* = 0.0043; ****p < 0.0001: sham group vs. CSCI group; ^####^*p* < 0.0001: CSCI group vs. CSCI with decompression group]. **(C)** Animals were tested weekly starting from 1 dpi using the IPT, and the angle (°) was recorded to reveal motor function recovery [Two-way ANOVA, *F*(6,112) = 4.861, *p* < 0.001; ****p < 0.0001: sham group vs. CSCI group; ^##^*p* = 0.0052: CSCI group vs. CSCI with decompression group]. **(D)** BBB scores for recovered and non-recovery rats after decompression at 3 wpi. The Sham group: *n* = 10; CSCI group: *n* = 10; CSCI with decompression group: *n* = 11. df: the degrees of freedom; Mean ± 95% CI.

Surgical decompression has been regarded as a safe treatment for incomplete CSCI (Bakhsheshian et al., 2017; Sakaura et al., 2019), which results in improved functional status and quality of life for patients with various diseases, including CSM, ossification of the posterior longitudinal ligament (OPLL), and spinal stenosis. Here, we used the BBB score and IPT to evaluate changes in motor function after surgical decompression in a rat model of CSCI (Figure 2A). The results showed that the motor function gradually decreased from 1 to 3 wpi after CSCI. However, rats with surgical decompression at 1 wpi exhibited significantly improved motor function compared to rats without decompression (Figures 2B,C). Moreover, two of eleven CSCI rats did not recover at 3 wpi in comparison with 1 wpi in terms of BBB score and over 80% of rats exhibited a significant recovery after early decompression in our model (Figure 2D).

### Structural and Cellular Changes and Hypoxic Level in the Decompressed Spinal Cord

**FIGURE 3:**
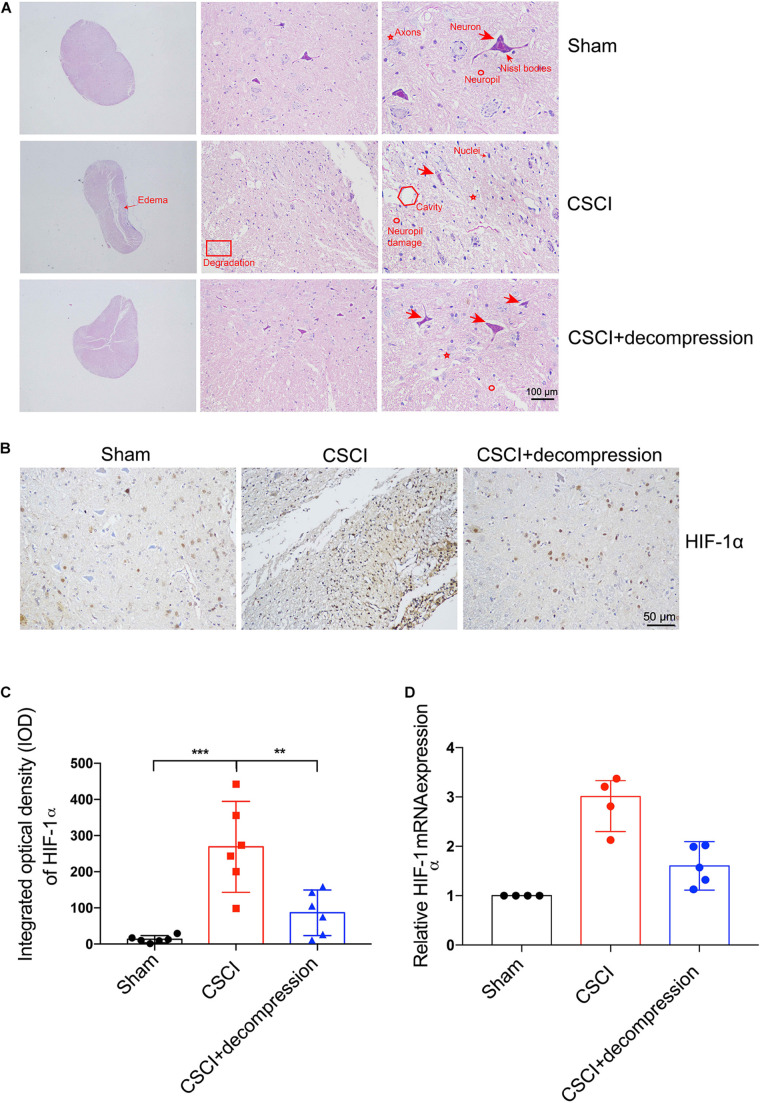
Decompression-protected neurons and improved hypoxia environment after CSCI. **(A)** HE staining of lesion sites from the sham, CSCI, and CSCI with decompression groups (20×, 200×, and 400× magnification; scale bar, 100 μm). **(B)** HIF-1α expression, as assessed by IHC (200× magnification; scale bar, 50 μm). **(C)** HIF-1α IHC staining, as quantitated by the IOD [One-way ANOVA, *F*(2,15) = 17.18, *p* < 0.001; Tukey *post hoc*: ***p < 0.001, **p = 0.003]. **(D)** Relative HIF-1α expression, as determined by RT-qPCR. HE staining and IHC: *n* = 6 each group; Mean ± 95% CI. RT-qPCR: the Sham group: *n* = 4; CSCI group: *n* = 4; CSCI with decompression group: *n* = 5; Medians ± interquartile ranges, the sample size was too low to perform statistics for these data.

Hematoxylin and eosin (HE) staining revealed histological changes at the injury epicenter under different levels of magnification (Figure 3A). The spinal cord was intact in the sham group. In the CSCI without decompression group, the compressed spinal cord exhibited structural damage, including fragmentation of neuronal nuclei, pyknosis, neuropil damage, degradation of the extracellular matrix, interstitial edema, cytoplasmic reduction, and cavity formation. Cellular changes were also observed at 3 wpi following CSCI, including neuron loss and axon degradation. Interestingly, differences were observed in the structural and cellular changes between the groups with and without decompression. After decompression, fragmentation of neuronal nuclei, degradation of the extracellular matrix, pyknosis, neuropil damage, interstitial edema, cytoplasmic reduction, cavity formation, neuron loss, and axon degradation were clearly alleviated.

HIF-1α IHC staining and RT-qPCR were performed to evaluate the hypoxic levels after CSCI (Figure 3B). As shown in Figures 3C,D, the quantitative analysis results indicate that HIF-1α expression in the spinal cord lesion site was increased after 3 weeks of compression. Preliminary data suggest that surgical compression at 1 wpi may improve the hypoxia environment and decrease HIF-1α expression compared with no decompression. However, further experiments are needed to confirm this notion.

### Expression of VEGF, vWF, MMP-9, and MMP-2 in the Decompressed Spinal Cord

**FIGURE 4:**
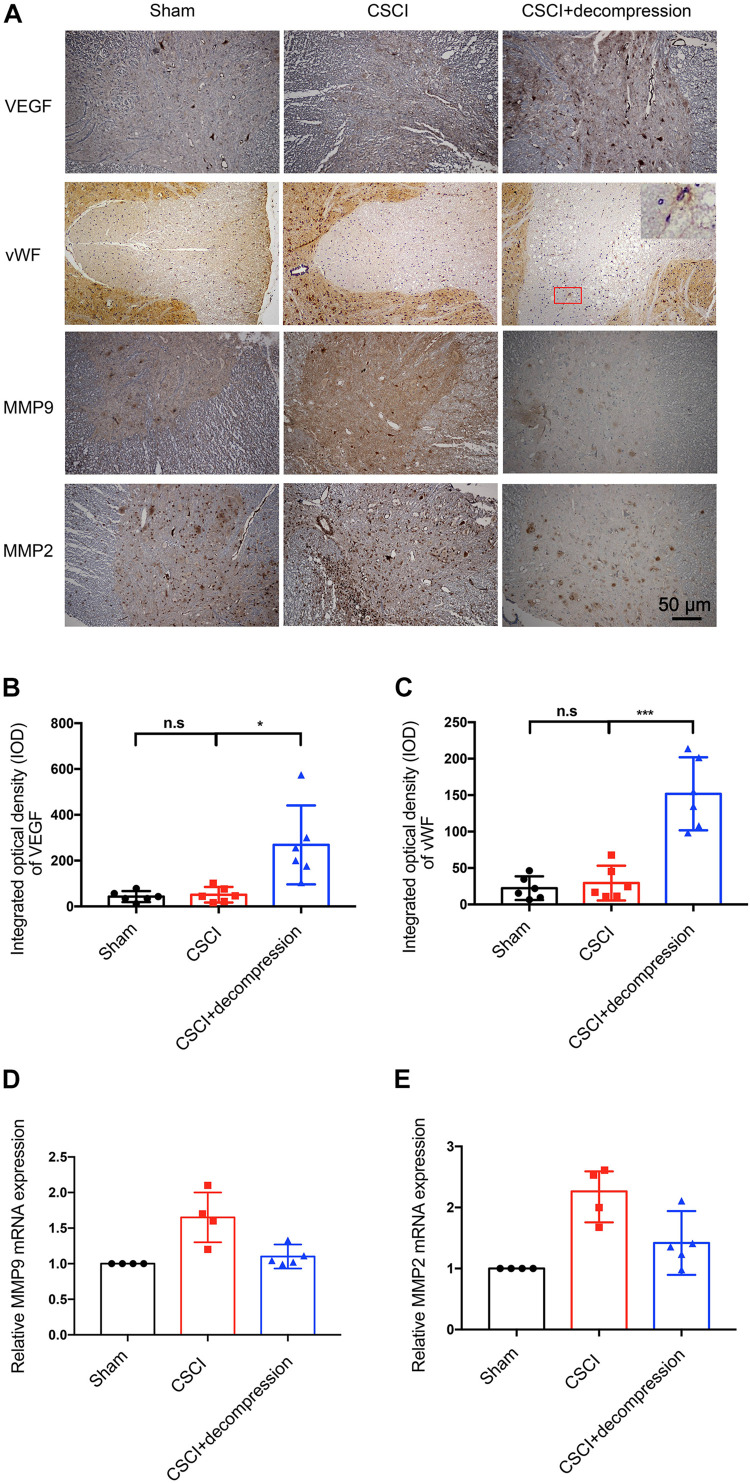
Decompression promoted VEGF and vWF expression and inhibited MMP-9 and MMP-2 expression. **(A)** VEGF, vWF, MMP-9, and MMP-2 expression was assessed by IHC (200× magnification; scale bar, 50 μm). IHC staining of **(B)** VEGF [One-way ANOVA, *F*(1.021,5.103) = 10.12, *p* = 0.024; Tukey *post hoc*: n.s.: not significant; *p = 0.045] and **(C)** vWF [One-way ANOVA, *F*(1.075,5.376) = 29.33, *p* = 0.002; Tukey *post hoc*: n.s.: not significant; ***p < 0.001] as quantitated by analyzing the IOD. Relative expression of **(D)** MMP-9 and **(E)** MMP-2 as assessed by RT-qPCR. IHC: *n* = 6 each group. Mean ± 95% CI; RT-qPCR: the Sham group: *n* = 4; CSCI group: *n* = 4; CSCI with decompression group: *n* = 5; Medians ± interquartile ranges, the sample size was too low to perform statistics for these data.

Vascular endothelial growth factor (DuBois and Demetri, 2007) and vWF (Randi and Laffan, 2017) have been reported as angiogenesis markers in many diseases, while MMPs such as MMP-9 and MMP-2 are related to inflammation and the BSCB (Na et al., 2015; Yao et al., 2018). Recently, many researchers have suggested that axonal degeneration, vascular dysfunction, and BSCB disruption may be key processes in neurological function recovery following decompression (Badhiwala et al., 2018; Blume et al., 2020). We previously reported on vascular and BSCB alterations as well as a correlation between MMP-9 and motor function recovery after CSCI, which indicated that angiogenesis and MMP may play critical roles in the process of motor function recovery (Long et al., 2014; Cheng et al., 2015, 2019; Xu et al., 2017). Therefore, we further investigated VEGF and vWF expression in spinal cord by using IHC to evaluate the potential effects of surgical decompression on angiogenesis. We also explored the effects of decompression on MMP-9 and MMP-2 (Figure 4). The results revealed that VEGF expression level in spinal cord gray matter and vWF expression level in spinal cord white matter were decreased, while MMP-9 and MMP-2 levels were increased in endothelial cells, neurons, and glia of the spinal cord after CSCI. Interestingly, VEGF and vWF levels were dramatically increased after decompression. Moreover, preliminary results suggest that decompression resulted in decreased MMP-9 and MMP-2 expression at 3 wpi (Figures 4B–E and Supplementary Figures S1A,B). However, further experiments are needed to confirm this notion. These data reveal that decompression may influence angiogenesis and BSCB function in the lesional spinal cord.

### Notch-1 Expression Was Up-Regulated and Positively Associated With Motor Function Recovery After Decompression

**FIGURE 5:**
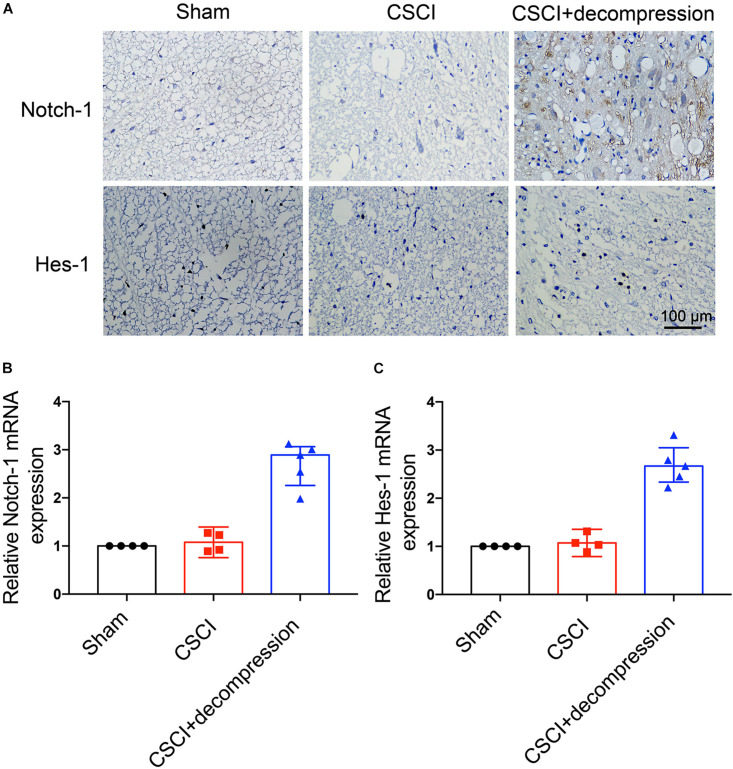
Decompression-induced up-regulated Notch-1 expression. **(A)** Notch-1 and Hes-1 expression was assessed by IHC (400× magnification; scale bar, 100 μm). **(B)** Relative Notch-1 expression and **(C)** relative Hes-1 expression, as assessed by RT-qPCR. IHC: *n* = 6 each group; RT-qPCR: The Sham group: *n* = 4; CSCI group: *n* = 4; CSCI with decompression group: *n* = 5; Medians ± interquartile ranges, the sample size was too low to perform statistics for these data.

**FIGURE 6:**
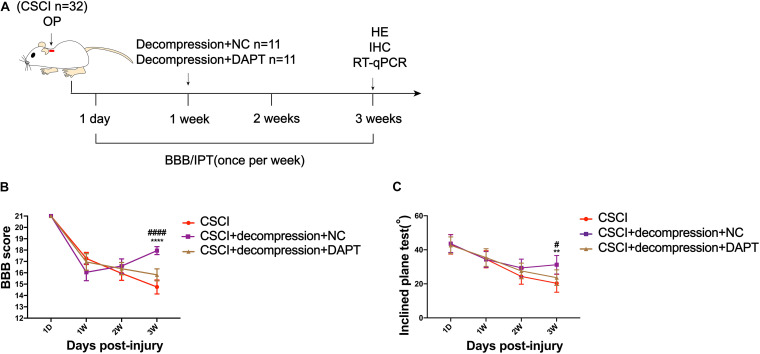
Inhibition of Notch-1 attenuated motor function recovery after decompression. **(A)** Experimental design. **(B)** Animals were tested weekly starting from 1 dpi in an open field, and motor function recovery was evaluated according to the BBB score [Two-way ANOVA, *F*(6,116) = 16.85, *p* < 0.0001; ****p < 0.0001: CSCI group vs. CSCI with decompression and NC group; ^####^*p* < 0.0001: CSCI with decompression and NC group vs. CSCI with decompression and DAPT group]. **(C)** Animals were tested weekly starting from 1 dpi using the IPT, and the angle (°) was recorded to reveal the motor function recovery [Two-way ANOVA, *F*(6,116) = 3.279, *p* = 0.0412; **p = 0.0052: CSCI group vs. CSCI with decompression and NC group; ^#^*p* = 0.0431: CSCI with decompression and NC group vs. CSCI with decompression and DAPT group]. The CSCI group: *n* = 10; CSCI with decompression and NC group: *n* = 11; CSCI with decompression and DAPT group: *n* = 11. Mean ± 95% CI.

To investigate whether Notch-1 signaling is activated after decompression, IHC and RT-qPCR were applied to detect the expression of Notch-1 and its downstream gene Hes-1. Preliminary data indicates that decompression lead to an upregulation of Notch-1 and Hes-1 expression levels (Figure 5 and Supplementary Figures S1C,D). However, further studies need to confirm these findings. These preliminary results indicate that the Notch-1 signaling pathway may play a role in motor function recovery after CSCI. Furthermore, we down-regulated Notch-1 expression by applying DAPT, a γ-secretase inhibitor, to deactivate the Notch-1/Hes-1 signaling pathway (Figure 6A). Figures 6B,C clearly show that the motor function recovery was inhibited following down-regulation of Notch-1 after decompression, as the motor function recovery differed from that of the control group at 3 wpi. These results reveal that Notch-1 up-regulation after decompression plays a critical role for motor function recovery in a rat CSCI model.

### Effects of Notch-1 Down-Regulation on Pathophysiological Changes in the Decompressed Spinal Cord

**FIGURE 7:**
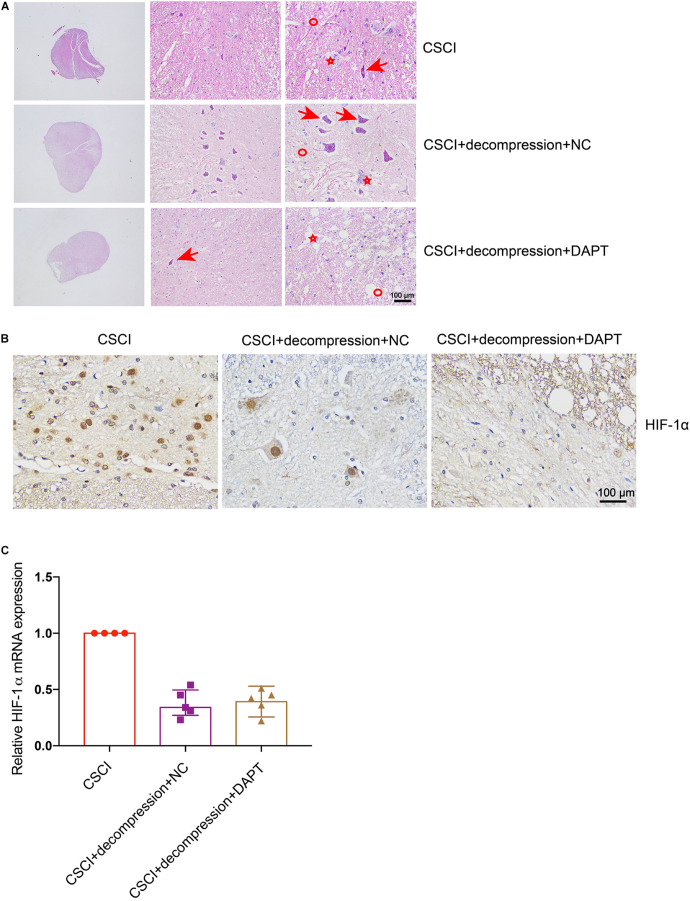
Inhibition of Notch-1 does not have neuron-protective effects on CSCI after decompression. **(A)** HE staining of lesion sites from the CSCI, CSCI with decompression and NC, and CSCI with decompression and DAPT groups (20×, 200×, and 400× magnification; scale bar, 100 μm). **(B)** HIF-1α expression was assessed by IHC (400× magnification; scale bar, 100 μm). **(C)** The relative HIF-1α expression was assessed by RT-qPCR. HE staining and IHC: *n* = 6 each group; RT-qPCR: the CSCI group: *n* = 4; CSCI with decompression and NC group: *n* = 5; CSCI with decompression and DAPT group: *n* = 5; Medians ± interquartile ranges, the sample size was too low to perform statistics for these data.

As previously described, HE and IHC staining were used to investigate pathological changes in our study. After the down-regulation of Notch-1, the fragmentation of neuronal nuclei, neuropil damage, interstitial edema, cytoplasmic reduction, cavity formation, degradation of the extracellular matrix, neuron loss, and axon degradation were more pronounced than in the group of CSCI without decompression (Figure 7A). However, the down-regulation of Notch-1 did not have any effect on the compression-induced hypoxia condition (Figure 7B and Supplementary Figure S2A). We were not able to detect any difference in our preliminary analysis of HIF-1α expression between the animals with or without DAPT treatment after decompression (Figure 7C). Further experiments are needed to confirm this result.

**FIGURE 8:**
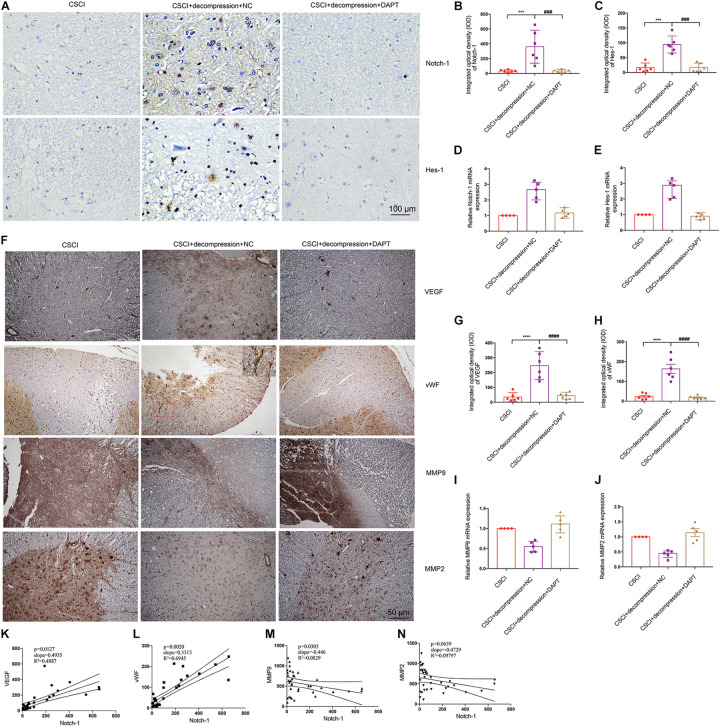
Inhibition of Notch-1 resulted in decreased VEGF, vWF, MMP-9, and MMP-2 expression. **(A)** Notch-1 and Hes-1 expression levels were assessed by IHC (400× magnification; scale bar, 100 μm). IHC staining of **(B)** Notch-1 [One-way ANOVA, *F*(2,15) = 14.12, *p* < 0.001; Tukey *post hoc*: ***p < 0.001, ^###^*p* < 0.001] and **(C)** Hes-1 was quantitated by analyzing the IOD [One-way ANOVA, *F*(2,15) = 30.64, *p* < 0.001; Tukey *post hoc*: ***p < 0.001, ^###^*p* < 0.001]. Relative expression levels of **(D)** Notch-1 and **(E)** Hes-1 were assessed by RT-qPCR. **(F)** VEGF, vWF, MMP-9, and MMP-2 expression levels were assessed by IHC (200× magnification; scale bar, 50 μm). IHC staining of **(G)** VEGF [One-way ANOVA, *F*(2,15) = 27.56, *p* < 0.0001; Tukey *post hoc*: ****p < 0.0001, ^####^*p* < 0.0001], and **(H)** vWF [One-way ANOVA, *F*(2,15) = 34.93, *p* < 0.0001; Tukey *post hoc*: ****p < 0.0001, ^####^*p* < 0.0001] was quantitated by analyzing the IOD. Relative expression levels of **(I)** MMP-9 and **(J)** MMP-2 were assessed by RT-qPCR. Correlation between Notch-1 expression and **(K)** VEGF, **(L)** vWF, **(M)** MMP-9, and **(N)** MMP-2. IHC: *n* = 6 each group; Mean ± 95% CI. RT-qPCR: the CSCI group: *n* = 4; CSCI with decompression and NC group: *n* = 5; CSCI with decompression and DAPT group: *n* = 5; Medians ± interquartile ranges, the sample size was too low to perform statistics for these data.

It was previously reported that activation of the Notch-1 signaling pathway can up-regulate VEGF, MMP-2, and MMP-9 by triggering the activation of NF-κB (Li et al., 2016). To further elucidate whether Notch-1 activation is associated with VEGF, vWF, MMP-9, and MMP-2 expression after decompression in the CSCI model, the expression of these factors was detected after Notch-1 down-regulation (Figure 8). Interestingly, following the inhibition of Notch-1, VEGF, and vWF expression levels were significantly lower compared with the control group (Figures 8G,H). In addition, preliminary data suggest that the down-regulation of Notch-1 increased MMP-9 and MMP-2 expression (Figures 8I,J and Supplementary Figures S2B,C), however, further experiments are needed to confirm these findings. The correlation among the expression of VEGF, vWF, MMP-9, MMP-2, and Notch-1 is presented in Figures 8K–N. The results demonstrate a significant correlation between the expression of Notch-1 and VEGF, vWF, and MMP-9.

Overall, the results indicate that early surgical decompression promoted motor function recovery may through protecting neurons and influencing the expression of angiogenesis-related markers and MMPs. Moreover, activation of the Notch-1 signaling pathway was shown to play an important role in motor function recovery following decompression.

## Discussion

Our study clarified the pathophysiological changes that occur in the spinal cord after decompression and demonstrated that early surgical decompression partially promoted motor function recovery in a water-absorbable polyurethane polymer compressive SCI. The motor function improvement achieved by surgical decompression was paralleled by improvements in structural and cellular damage, increased angiogenesis markers (VEGF and vWF), and reduced MMPs (MMP-9 and MMP-2) in the decompressed spinal cord. Moreover, the Notch-1/Hes-1 signal pathway was found to play an important role in the prognosis following decompression.

Due to the lack of a proper spinal cord compression model, pathophysiological changes following decompression are difficult to assess in rodent CSCI models (Akter et al., 2020). A stable, reusable material should also be considered. We therefore characterized rats with a chronic C6 compressive injury by performing IPTs and assessing BBB scores in an open field. The material used in this work gradually expanded after implantation, and therefore, the injured animals developed significant motor dysfunction after 1 wpi. The behavior outcomes in our cervical CSCI model are in line with growing evidence that rats begin to develop motor dysfunction after 1 week post-compression (Wang et al., 2012; Dhillon et al., 2016). Therefore, our study can partially reflect the sub-acute injury process of CSCI. These results indicate that water-absorbable polyurethane polymer may be a suitable material for imitating the occurrence and development of CSM in patients.

In the current study, surgical decompression was able to partially improve the motor function recovery. The pathophysiological mechanisms governing the effects of decompression are complicated. In clinical practice, outcomes are also influenced by the compression degree and symptom duration of each patient. Improvements in structural and cellular changes in the decompressed spinal cord may play a role in the progress of motor function recovery. It has been well-documented that compression-induced neuron loss is paralleled by motor dysfunction (Ogino et al., 1983; Yu et al., 2009). Many factors such as cell death, inflammation, and ischemia can lead to neuron loss (Akter et al., 2020). Tanabe et al. (2011) suggested that HIF-1α may be increased in the twy/twy model and that hypoxic stress may promote neuronal cell death. However, ischemic-hypoxic changes in CSCI have not been widely investigated (Akter et al., 2020). Our data show that hypoxia conditions, which are also associated with motor function recovery, were improved after surgical decompression.

In addition, we observed that angiogenesis markers, i.e., VEGF and vWF, were increased following decompression. It has been reported that vascular dysfunction and angiogenesis may affect myelin damage, glial fibrosis, and necrosis after cervical spinal cord compression (Gooding et al., 1975; Ng and Lee, 2019). Furthermore, reduced blood flow to the spinal cord is associated with vascular dysfunction after compression (Franklin and Ffrench-Constant, 2008; Kurokawa et al., 2011). The molecular mechanisms of angiogenesis in decompression remain unclear. We also found that decompression can significantly decrease MMP-9 and MMP-2 expression. MMPs are associated with BSCB dysfunction (Zhang et al., 2017), and a similar expression pattern in CSM was described by Karadimas et al. (2013b). We previously found that the BSCB is disrupted after long-term spinal cord compression (Xu et al., 2017). Therefore, the high expression levels of MMP-9 and MMP-2 in the lesional spinal cord suggest these factors may play a role in BSCB disruption and that decompression may improve the BSCB by decreasing MMP-9 and MMP-2 expression. However, further research is needed to confirm this interpretation.

Notch signaling in CNS diseases is associated with cell death, angiogenesis, and BSCB function (Fassbender et al., 2011; Cai et al., 2016). Recently, studies have indicated that Notch-1 signaling can modulate VEGF, MMP-9, and MMP-2 expression *in vitro* and *in vivo* (Li et al., 2016). In our study, Notch-1 and its downstream gene Hes-1 were decreased under compression and increased after decompression. More interestingly, the down-regulation of Notch-1 can inhibit angiogenesis marker expression and promote MMP-9 and MMP-2 expression. The role of Notch-1 in angiogenesis is still unclear. Tang et al. (2018) reported that inhibition of the Notch-1/DLL4 pathway promotes angiogenesis in rats, while (Gao et al., 2012) demonstrated that Notch-1/HIF-1α interactions mediate hypoxia-induced angiogenesis in inflammatory arthritis. More investigations are needed to clarify these findings. In our study, HIF-1α expression in the decompressed spinal cord was not influenced by the inhibition of Notch-1. Nevertheless, the improvements in structure and cellular changes were weakened after Notch-1 inhibition. Thus, the underlying mechanisms require further investigation.

The dipeptidic compound DAPT (N-[N- (3,5-difluorophenacetyl) -L-alanyl] -S-phenylglycine t-butyl ester), a γ−secretase inhibitor was tested *in vivo* in Dovey et al. (2001). γ-Secretase displays poor substrate specificity (Panza et al., 2010) and (Takeshita et al., 2007) suggested that γ-secretase can mediate Notch1 cleavage and activation by phosphatidylinositol 3-kinase/Akt pathway in postnatal angiogenesis. However, it still needs to be explored whether other signaling molecules/pathways besides Notch-1 may be affected in our CSCI model. In a transgenic mouse model of Alzheimer’s Disease, it was demonstrated that DAPT could reverse contextual memory deficit through targeting beta-amyloid (Comery et al., 2005). It has also been reported that DAPT could inhibit all four Notch receptors and is a promising option for cancer therapy (Shih and Wang, 2007). Moreover, the γ-secretase inhibitor DAPT could decrease angiogenesis and inhibit VEGF-induced endothelial cell proliferation, migration, and survival as well (Takeshita et al., 2007).

To the best of our knowledge, this study is the first to focus on the influence of early surgical decompression on motor function recovery and physiological and pathological changes in CSCI rats. Our findings can serve as a basis for future studies in rodents to explore the molecular and cellular changes underlying motor dysfunction after CSM and decompression-induced improvements.

Nevertheless, our study has several limits. It has been reported that surgical decompression for the treatment of CSM is associated with not only motor function recovery, but also sensory function recovery (Fehlings et al., 2013; Zaveri and Jaiswal, 2019). However, the sensory function was not evaluated in this study. In addition, we found that the inhibition of Notch-1 was associated with the expression of angiogenesis markers and MMPs, but the underlying mechanisms governing these changes were not pursued. Thus, the manner in which Notch-1 modulates the expression of these factors after decompression remains unclear. The NF-κB signaling pathway can be triggered by the activation of Notch-1; therefore, this pathway can be initiated by up-regulated expression of NF-κB target genes, including VEGF, MMP-9, and MMP-2 (Karadimas et al., 2013b). Hence, it would be valuable to explore whether the NF-κB signaling pathway is activated by Notch-1 after decompression in a rat CSCI model. Further research is also needed to demonstrate that activation of Notch-1 is sufficient to induce the protection. Furthermore, although we demonstrated the changes in angiogenesis markers and MMPs in the decompressed spinal cord via histological approaches, the detection of angiogenesis and BSCB disruption via morphological and functional approaches is needed in future work. Given that the sample size in different groups of this exploratory study is not large enough, therefore it is necessary to increase the sample size to further explore the underlying mechanisms of our preliminary findings.

In summary, our study illustrated the preliminary physiological and pathological changes occurring in the compressed and decompressed spinal cord in the sub-acute injury process of CSCI rats. In addition, we confirmed that early surgical decompression can partially improve motor function recovery. Our data indicate that activation of the Notch-1 signaling pathway may play a role in the prognosis of CSCI after decompression, which may provide a new drug approach in order to enhance recovery following surgery.

## Data Availability Statement

The raw data supporting the conclusions of this article will be made available by the authors, without undue reservation.

## Ethics Statement

The animal study was reviewed and approved by The first affiliated hospital of Sun Yat-sen University.

## Author Contributions

XC analyzed the data, wrote the manuscript, and created the figures. ZY performed the experiments and analyzed the data. JX performed the experiments. DQ revised the manuscript and designed the experiments. HL designed the experiments, contributed to manuscript writing, and gave final approval of the manuscript. All authors contributed to the article and approved the submitted version.

## Conflict of Interest

The authors declare that the research was conducted in the absence of any commercial or financial relationships that could be construed as a potential conflict of interest.
