# Corrigendum: Pathophysiological Changes and the Role of Notch-1 Activation After Decompression in a Compressive Spinal Cord Injury Rat Model

**DOI:** 10.3389/fnins.2021.665669

**Published:** 2021-02-25

**Authors:** Xing Cheng, Zhengran Yu, Jinghui Xu, Daping Quan, Houqing Long

**Affiliations:** ^1^Guangdong Provincial Key Laboratory of Orthopaedics and Traumatology, Orthopaedic Research Institute/Department of Spine Surgery, The First Affiliated Hospital, Sun Yat-sen University, Guangzhou, China; ^2^PCFM Lab, GD HPPC Lab, School of Materials Science and Engineering, Sun Yat-sen University, Guangzhou, China

**Keywords:** decompression, CSCI, motor function, pathophysiological changes, Notch-1

In the published article, there was an error in affiliation “1”. Instead of “Department of Spine Surgery, The First Affiliated Hospital, Sun Yat-sen University, Guangzhou, China”, it should be “Guangdong Provincial Key Laboratory of Orthopaedics and Traumatology, Orthopaedic Research Institute/Department of Spine Surgery, The First Affiliated Hospital, Sun Yat-sen University, Guangzhou, China”.

The authors apologize for this error and state that this does not change the scientific conclusions of the article in any way. The original article has been updated.

