# Supplementary figures and images for: Pathophysiological Changes and the Role of Notch-1 Activation After Decompression in a Compressive Spinal Cord Injury Rat Model
Source: Front Neurosci. 2021 Jan 28;15:579431. doi: 10.3389/fnins.2021.579431 (PMC7876297; doi:10.3389/fnins.2021.579431)

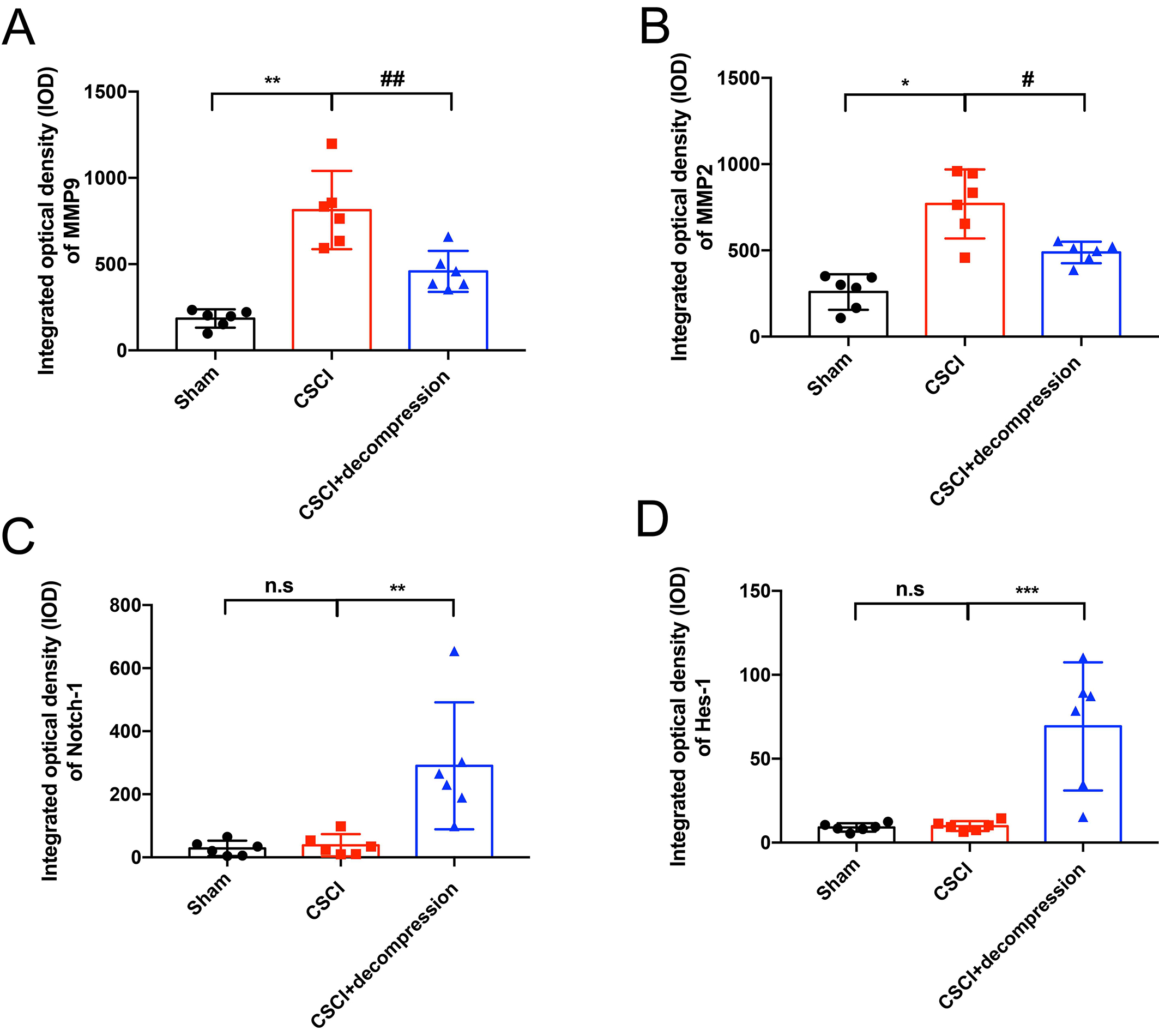

Supplement: Supplementary file 1 [file Image_1.JPEG]

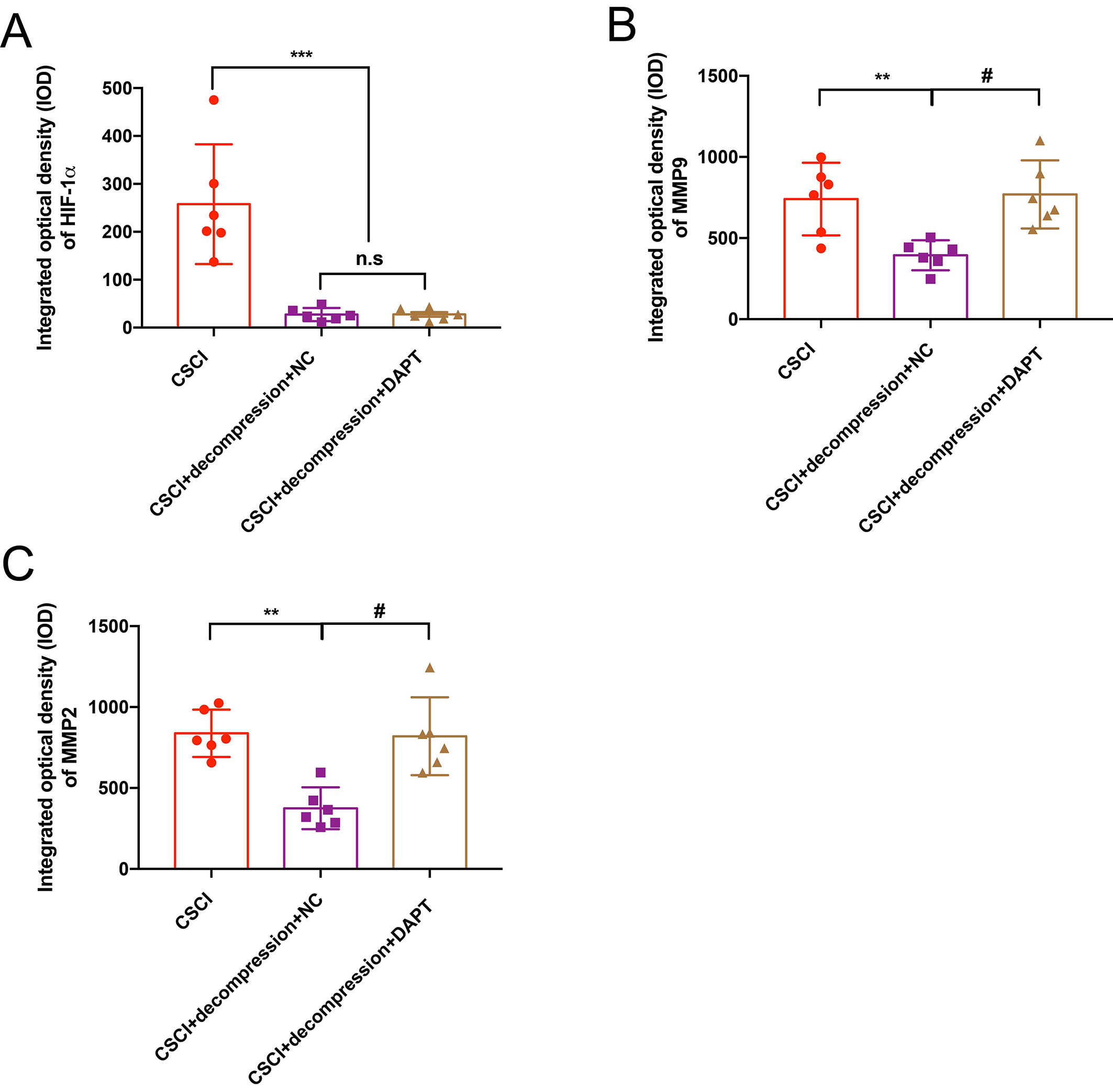

Supplement: Supplementary file 2 [file Image_2.JPEG]
