# Supplementary material for: Pathophysiological Changes and the Role of Notch-1 Activation After Decompression in a Compressive Spinal Cord Injury Rat Model
Source: Front Neurosci. 2021 Jan 28;15:579431. doi: 10.3389/fnins.2021.579431 (PMC7876297; doi:10.3389/fnins.2021.579431)
Supplement: Supplementary file 4 [file Table_2.DOCX]

**Supplementary information**

**Table S1. Primers used for RT-qPCR**

| Gene | Forward primer (5'-3') | Reverse primer (5'-3') |
| --- | --- | --- |
| U6 | CTCGCTTCGGCAGCACA | AACGCTTCACGAATTTGCGT |
| GAPDH | CAAGGTCATCCATGACAACTTTG | GTCCACCACCCTGTTGCTGTAG |
| Notch-1 | CTGGTCAGGGAAATCGTG | TGGGCAGTGGCAGATGTAG |
| Hes-1  HIF-1α | ACACCGGACAAACCAAAGAC  TATGAGCCAGAAGAACTTTTAGGC | AATGCCGGGAGCTATCTTTC  CACCTCTTTTGGCAAGCATCCTG |
| MMP-9 | ATCCAGTTTGGTGTCGCGGAGC | GAAGGGGAAGACGCACAGCT |
| MMP-2 | CTCAGATCCGTGGTGAGATCT | CTTTGGTTCTCCAGCTTCAGG |


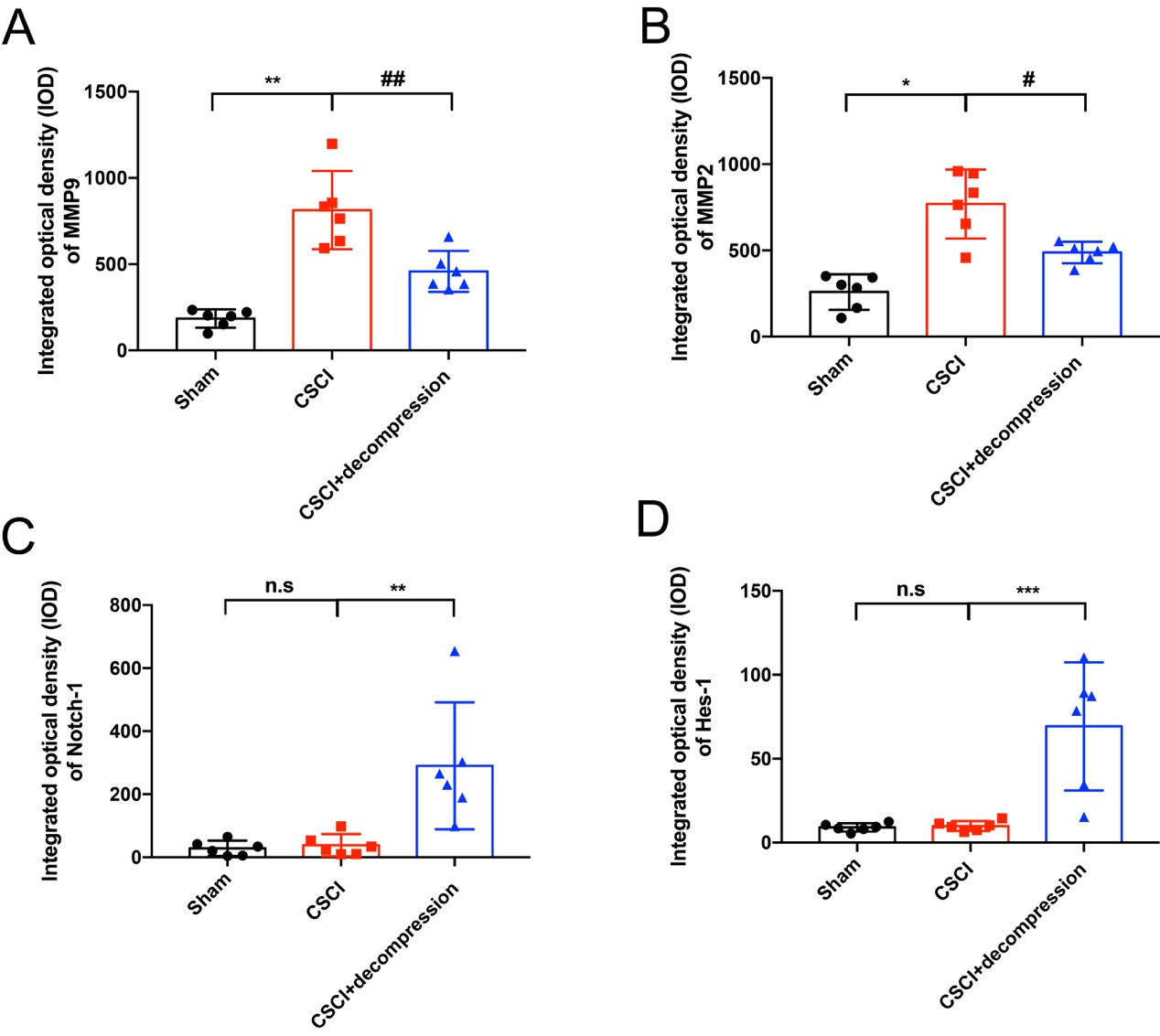
**F****igure S1. The expression of** **MMP-9, MMP-2, Notch-1 and Hes-1**

IHC staining of (A) MMP-9 (One-way ANOVA, F(1.154, 5.769) = 21.31, p = 0.004; Tukey post hoc: **p = 0.002, ^##^p = 0.005), (B) MMP-2 (One-way ANOVA, F(1.362, 6.812) = 18.24, p = 0.003; Tukey post hoc: *p = 0.012, ^#^p = 0.044), (C) Notch-1 (One-way ANOVA, F(2, 15) = 10.33, p = 0.002; Tukey post hoc: n.s.: not significant, **p = 0.004) and (D) Hes-1 (One-way ANOVA, F(2, 15) < 0.001, p = 0.002; Tukey post hoc: n.s.: not significant, ***p < 0.001) as quantitated by analyzing the IOD. IHC: n=6 each group. Mean ± 95% CI.


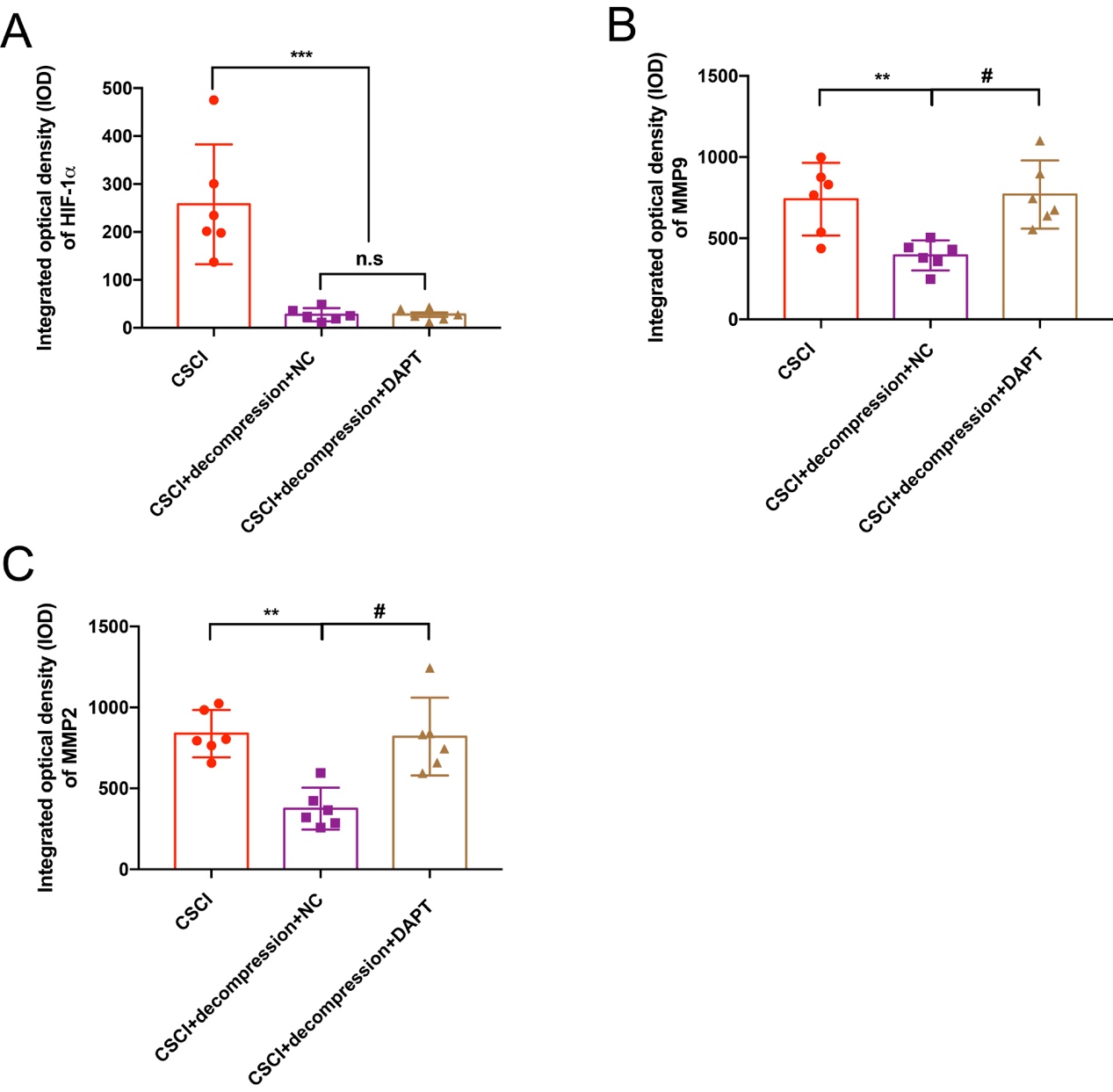


**Figure S2. The expression of** **HIF-1**α, **MMP-9 and MMP-2**

IHC staining of (A) HIF-1α (One-way ANOVA, F(2, 15) = 21.98, p < 0.001; Tukey post hoc: ***p < 0.001; n.s.: not significant), (B) MMP-9 (One-way ANOVA, F(1.167, 5.837) = 8.72, p = 0.024; Tukey post hoc: **p = 0.004, ^#^p = 0.023) and (C) MMP-2 (One-way ANOVA, F(1.447, 7.233) = 11.82, p = 0.007; Tukey post hoc: **p = 0.005, ^#^p = 0.015) as quantitated by analyzing the IOD. IHC: n=6 each group. Mean ± 95% CI.
